# Adhesive Gold Foil—Its Discovery, History, and Use

**Published:** 1870-03

**Authors:** A. Westcott

**Affiliations:** Syracuse N. Y. Formerly Professor of Operative and Mechanical Dentistry in the Baltimore College of Dental Surgery, and late Professor in the New York College of Dental Surgery.


### ARTICLE VI.

### *Adhesive Gold Foil—Its Discovery, History, and Use.*

By A. Westcott, M.D.,D.D.S., Syracuse N. Y.

Formerly Professor of Operative and Mechanical Dentistry in the Baltimore College of Dental Surgery, and late Professor in the New York College of Dental Surgery.

There is perhaps no one principle connected with dental operations of greater moment, or whose value is more universally acknowledged by the profession, than the *adhesiveness of gold*, either as foil, sponge, or however gold may be prepared, to make this principle available in filling teeth. Before this principle was discovered, the theory of making fillings permanent was, to a large extent, that they were kept in by leaving the orifice through which the gold is inserted smaller than the cavity within, and they were thus "dove-tailed" into the tooth. I have now in mind an advertisement issued *more than thirty years ago*, by a dentist who now stands at the very head of the profession, as an operator, which read substantially as follows: "I insert my fillings in such a manner that they never come out, which I do by making the cavity *inside* much larger than the orifice through which the gold is inserted, thus giving it a firm mechanical holding." Now, this was a good, frank avowal of his way and manner of operating, and showed that he was then, what he has ever since proved himself to be, *an honest man.* This was, moreover, a *taking* advertisement; for in a very short time this young man was doing a fine business in a small country village, where, previously, dentistry had hardly been heard of and I have to confess that I looked upon this representation myself with no little admiration.

I have for a very long time been intending to prepare a paper upon *the origin and history of adhesive gold foil,* but have been waiting for the legion who have from time to time laid claim to originality in the discovery of this great dental fact,—that gold is susceptible of adhering to itself, or of being virtually welded at common temperatures,—to write out their respective claims, feeling that when all the rest were through, I would bring up the rear. How far or how perfectly I have thus been outflanked I shall let dates and facts speak for themselves.

In the spring of 1840, I was practicing dentistry in Homer, Cortland Co., N. Y., and ordered from T. B. Fitch, Esq., then a druggist at Syracuse. now an eminent banker of this city, and who well remembers the circumstance, an eighth ounce of gold foil by *mail.* It being in the days of high postage, and Mr. F. knowing that my extra pennies were few, undertook to do me the favor of economizing for me, by removing the intervening papers from between the leaves of foil. I received the foil in this way, and to my surprise, and I may well say, dismay, I was wholly unable to separate the leaves of foil from each other. They had, by some magic which had never before come to my notice, literally "grown together." What was to be done? I was not only *foiled* in regard to my promised operations, but I must await the going and coming of the slow coach, the long distance of thirty miles, before I could take another step, and meanwhile writhe under the uncertainty of being able to better myself after all. More than this, I was in danger of suffering (for me) a heavy pecuniary loss, unless I could convince Mr. Fitch that the responsibility was upon his shoulders, in consequence of the blunder which he had committed, in the removal of the aforesaid papers. Here was presented to my mind the triple misfortune of the probable loss of my "job," the actual loss of two or three day's time, and the possible loss of a whole eighth ounce of gold foil!

This was surely a time for meditation and reflection, and I did reflect. This may possibly provoke a smile from some reader, but it must be from some one who did not begin the world as I began it. In all this there was to me "no levity." But in my sad ruminations I did not allow myself to forget the necessity of prompt action. I immediately wrote a sharp letter to Mr. Fitch, which was not mollified with a single expression of gratitude for his well-meant effort to do me a kindness, asking him immediately to replace the foil, by sending, "per first stage," another parcel, and with the strong injunction, to *leave the papers where he found them.* All this was as new to him as to me, and at once falling in with my suggestion, that "there must be something wrong about the *foil*," he immediately wrote to Mr. Barret, of Albany, who made the foil, for an explanation. As soon as the then slow mails could bring it he received a reply from Mr. B., which he lost no time in forwarding to me, apologizing to Mr. F. for having sent him such a batch of foil, and adding that, "once in awhile, foil would prove *sticky*, but he never sent out such foil when he knew it,"—all of which would have been very consoling to me had it accompanied the original package of foil sent me by Mr. Fitch.

But this information, "for immediate use," came too late. My reflections, ere it came, had taken another and very different channel. As soon as the shock was over, I began to inquire into the probable cause of this result, and as to whether it did not present an idea which might be made available. The question soon arose,—*If gold foil might be thus virtually "welded" in a mail bag, why not in the cavity of a tooth?*

I lost no time in putting this question to a full practical test. I began my experiments by screwing together two plane surfaces of ivory, and drilling a hole at their joining. so that when taken apart one-half of the hole would be in each piece. I could in this way easily remove my filling packed into this cavity. This I did repeatedly, till, by hammering, and otherwise testing these filling, I became perfectly satisfied that I could make, of any number of pieces or pellets, *one solid*, *integral piece* that would take and retain a polish under any circumstances; and I can here truthfully add, that the first filling in the mouth which I did with this foil, was the first perfectly polished filling that I had ever seen. Thus was my vexation turned into a joy, "far more exceeding;" and before Mr. Barrett's letter reached me, which I think was in about ten days, I was ready to contract for all the "sticky" foil I could get; and the moment I got his letter, addressed to Fitch, I wrote him, (B.) asking him to save all of his "sticky" foil for me; and I continued to get such foil of him, from time to time, while he remained in business, or till Watts found a method of producing such foil with certainty and uniformity. After satisfying myself that the adhesiveness of foil could be relied upon, the next question which arose was, What were the conditions necessary to secure it? or, on what principle was it affected? I became satisfied that the cohesion or adhesion (for these terms, for all practical purposes, are synonymous) was something more than an interknitting of particles,—it was a surface joining, and was none other than that *cohesion* exhibited by two lead bullets when two fresh-cut surfaces were pressed together, the two becoming almost inseparable.

Looking upon the phenomenon as simply cohesion, I at once concluded that the rule laid down in all works on natural philosophy, in order to secure cohesive attraction, must be observed, viz: that "the particles, or bodies to be made coherent, must be brought in *actual contact.*" In other words, the foil must be positively free from *all* foreign substances. Any one will find, by trying the experiment, that in the case of the lead balls, above referred to, that if he simply draw a finger over the fresh-cut surfaces, it will effectually break up their otherwise strong tendency to cohere. Foil, then, even when of the "sticky" variety, must be kept perfectly free from *every* foreign substance, such as dust, soil, and especially from moisture. How frequently is the operator first notified of unseen moisture by the fact that his pellets won't adhere? And he might as well dip them into oil, as in water or saliva, if he wishes to avail himself of this quality of adhesiveness. From such facts and considerations comes the rule,—*Keep your foil positively dry during your entire operation.*

How operators have acquired prominence by submarine fillings, I have yet to learn. I have always supposed, however, that their pluggers were made of a goose-quill or other *pen*, and I fancy that such is not the kind of greatness to be marred by distance. To say the least, I do not believe that a *close* inspection of such fillings ever produced much "enchantment." Why adhesive foil loses this quality, simply by keeping, and why heating restores it, are questions which must have occurred to all. Is not the former question chiefly answered by supposing that it absorbs moisture, and the latter, that this humidity is dissipated by the heat?

But, to return more directly to the subject of this paper; I will say that from that day till the present time I have used adhesive foil in greater or less quantities, and with various modifications, till my present practice varies very materially from my early experience and practice with such foil. My discovery of, and experiments with, adhesive foil I immediately communicated to such dentists as I chanced to meet; for it will be understood that they were not as plenty in 1840 as in 1870, and the *adhesive principle* was as little understood and practiced among themselves, as was this principle or its application to gold, in filling teeth. It will also be born in mind, that at that time there was but one dental journal published in the United States, and that but just started. I think that the first article of mine which was ever published in a dental journal (and this was not written for publication) was my essay on "Dental Caries," read before the American Society of Dental Surgeons, in July, 1843, and published the following September. It will be thus seen why I did not immediately *publish* the result of the *accident* above alluded to.

I very soon discovered that to work such gold foil to best advantage I must modify my instruments, and different experiments very soon (within a few weeks) brought me to the conclusion that serrated points were, better than any others, calculated to work it with ease and dispatch, and I accordingly altered all of my pluggers to conform to this idea. These were the first set of the kind which I had ever seen or heard of. Perhaps this paper may meet the eye of some one who can antedate me. This is certainly possible, and would in no wise detract from the originality of *my* discovery. Nor would it be the first time I have myself been caught in a similar trap. I have sent many a supposed invention to the Patent Office, to be told that the invention was older than him who claimed it. My communications upon the subject have hitherto been oral, partly for the reasons already given, and partly because I have never happened to get about it. And even now, my writing this paper is about as purely accidental as was the discovery cf which it treats.

But notwithstanding I have not *written* anything upon this topic, I have never friled to talk upon it with every dentist who has expressed any interest in it. My theory and practice have ever been, that no *professional* has any right to withhold any fact or process from a professional brother, which would be useful to him, and which pertained to their common profession, nor to tie up such information by a *patent.* I have procured, within the last twenty years, about thirty patents for my own inventions, but no one of them has, in the remotest manner, pertained to the profession of dentistry in *any* of its branches, notwithstanding I can claim to have furnished, both to the operating-room and laboratory, many things and appliances which I knew to be patentable. Indeed, the discovery about which I am now writing was a most proper subject for a very valuable patent. " Dow, Jr.," wrote what he called " patent sermons," but I never heard of his being a communicant in good standing in any *orthodox* church.

From 1840 to 1846, inclusive, I probably conversed with one hundred or more dentists upon the subject of this paper and freely communicated to each and all every fact which I now set forth. In 1846 I became connected with the Baltimore College of Dental Surgery, as Professor of Theory and Practice, and gave a lecture upon this topic before the students in February 1848. Having given notice to Professor Harris that I was to give such a lecture, he invited all the dentists of the city* to be present, and many of them were present. While I was discussing this special topic, if I could not discern most plainly upon the countenances of some of them the smile of derision, it was clearly that of unbelief. The " welding of gold," at common temperatures, seemed an absurd not to say ridiculous doctrine.

Among the gentlemen present at that lecture, was the late Dr. E. Townsend, of Philadelphia, with whom I had previously conversed upon this subject, and who had expressed a deep interest in it. He expressed strong desire to see this principle demonstrated by one who had had some experience in the matter, and gave me a pressing invitation to spend a day or two with him, at his house in Philadelphia, on my way home. To this I agreed, and instead of spending " a day or two," I spent a full week with him, and filled teeth each day from two to four hours. My first patient was the doctor himself. I filled a compound cavity for him, in an

* **Baltimore,**

upper molar, in which I put nine sheets No. 4 foil, which filling he carried with him to his grave. *

During each of these sessions there were present, on his invitation, several dentists to see (to use his own language) "Dr Westcott's new method of using gold foil." I have been a little particular in recounting these circumstances, as his reticence in regard to myself, in a paper which he read some years afterward upon this subject, might not seem to tally with these statements. I am unwilling to think that he intended to do me injustice, but simply forgot to mention my name in that connection. When I went to his office, as above mentioned, he had not a single plugger with serrated points, and he for the first time and under my directions altered several to meet this end. I doubt not that there are several gentlemen still in practice who can fully corroborate the statements made above, and if this paper should chance to meet the eye of either of them, I should be much obliged if they would communicate with me upon the subject.

From that day to this I have used "sticky foil," and, as I have already said, with various modifications, till my present practice varies widely from that first adopted with such foil. Now, for the sake of comparing notes with other practitioners, or to enable them to compare their practice with mine, and not for the sake of advocating any special practice, as the *ne plus ultra*, or of raising a standard which no one need aspire to, by some different mode, I will describe briefly my present manner of using gold foil; for, among the different discoveries which I have made, is that *many others* bring out just as good operations as I can, by my method of filling teeth, by modes and manipulations which would prove a perfect failure in *my* hands. But while I

---

* Since the preparation of this paper, in looking over my correspondence with Dr. Townsend, I find in one of his letters, dated July 9th, 1847, the following playful allusion to this filling. In alluding to a "Mr· Lawrence" as the inventor of an appliance for holding napkins, he says: "You must recollect him as one of our party who stood over you at 'Broad Street House,' and saw you place that beautiful *door-plate* in my mouth which still gives out its golden lustre as brightly as at first." The doctor's allusion to this filling as a "*door-plate*," not only had reference to its great size, but its covering the whole anterior surface of the first upper molar, forward of which one or more teeth had been extacted: it could be seen at a considerable distance, whenever he opened his mouth, or even in ordinary conversation.

condemn no one's method, I may be allowed to prefer some one practice as having, in my judgment, advantages over others. Formerly, or in the early stages of my practice with adhesive foil, I used it to fill the entire cavity, whether great or small, but now make the following modifications:

1st. I seldom or never now fill a cavity entirely with adhesive foil.

2nd. I now use from eight to ten times as much *soft* foil, on the average, as I do adhesive foil.

3d. *I uniformly use both kinds in filling the same cavity*, unless it be a very small cavity, in which case I generally use adhesive foil only.

4th. *I uniformly use my soft foil in the form of cylinders*, varying in length, diameter, and compactness to suit the circumstances of the case.

5th. In each and every case I continue to use cylinders so long as I am sure that every part of the cavity can be reached by them, and they can be made as solid and secure as if done by pellets of either kind of gold.

6th. After the operation is carried as far as it can be with *certainty*, with cylinders, I then use firm but small-pointed instruments (pluggers) *with but little taper*, and pierce any and every part of the filling, and treat these permeations as new or original cavities. *These I fill with adhesive foil*, and this is now the only way or manner in which I use adhesive foil.

There are not a few good operators who have strong prejudices against the use of cylinders, but I am quite sure that their want of success in their use grows rather out of their want of practice with them, than from any inherent objection to their use. This may also arise from making them from improper foil, or from not knowing how to prepare and vary them so as to use them with certainty and ease.

In regard to the kind of foil of which cylinders should be made, it is a matter of the greatest moment that it be *soft* foil, in the strictest sense of the term. The most practiced hand will fail to do perfect work, if the operator is not careful to secure foil which is *positively* soft. In regard to the size, length, etc. of these cylinders, common sense dictates that they must be varied to suit the size, shape, and depth of the cavity in which they are to be used; but one precaution must not be overlooked, viz;, *they must not be too large.* Better by far err in the opposite direction,—using more time and smaller cylinders. In any given operation we begin with a cylinder as large as can with certainty be introduced and perfectly packed, and continue by using them smaller and smaller, till they can no longer be inserted without crushing. They must never be so large as to crush before reaching the bottom of the cavity into which they are to be thrust, and when this can be no longer done with *certainty*, then is the time to stop the use of cylinders and begin with pellets of adhesive foil. In large cavities we shall, by adopting this rule, use from one-sixth to one-eighth as much adhesive as we have used of soft foil.

Next, in regard to the *consistency* of these cylinders. *They must not be too hard.* Operators, unless constantly on their guard, are apt to roll their cylinders too tight, and hence make them too unyielding. They are tempted to do this to facilitate their introduction, when they are to meet with a resistance which would crush them if made softer. But this *spiking* sort of operation will not do. No cavity was ever perfectly filled with *unyielding* cylinders or round rods. These cylinders must be sufficiently soft to adapt themselves readily by lateral pressure to any and every irregularity of the cavity, and when we cannot introduce cylinders of this character into any required place, then is the time to change off for pellets. With *any* cylinders, and especially in large cavities, it not unfrequently becomes necessary to *alternate with pellets*, in order to secure some point too small to be fully reached by gold in the cylinder form. After a cylinder is carried to its destination and fully packed, I hardly need say that it must never be allowed to stir from its bed. To overcome this difficulty, especially in *large and shallow cavities*, it often becomes necesssary to use the left hand as an assistant to the right hand,—the office of the plugger in the left hand being mainly to hold the foil already packed in the place, while more foil is being introduced and packed by the plugger in the right hand. There is no difficulty, with a little practice, in thus using the left hand in conjunction with the right hand, even packing with both at the same instant; and I take this occasion to urge all young operators, before their notions and manipulations become stereotyped, to *practice using both hands at the same time*, or at least learn to do so, and thus be ready to meet any emergency that may call for it.

I will now offer some of the reasons which have influenced me to adapt my present method of filling teeth as above described. My first and strongest reason is, that in a particular class of cases *I can reduce my operations to far greater certainty* than by any method which I have hitherto adopted. I refer to approximal and lateral cavities, and especially those occurring in bicuspid and molar teeth. The critical point in filling such cavities is, to fill perfectly (if upper teeth) the upper half or portion of the cavity, so that it may be left with a certainty that this portion of the filling is perfect and that it may be finished flush with the edges of the walls of the cavity. This done, and the lower half or portion is easily managed. But if there is the least imperfection about the upper margin, we can never return to it with any hope of afterward making it perfect. This is one of those bills that admits of no amendments short of striking out the enacting clause. In other words, whenever an operator finds, on examining such a filling, that its upper portion is imperfect, he may as well at once remove the filling and begin it anew.

In such cases I use as my first cylinder, one which, when fully compressed, will fill from one-third to one-half of the cavity, and which is thrust upward against the upper shoulder or wall; meanwhile, when necessary, holding it from any outward thrust by an instrument in the left hand.

After it is fully solidified by direct upward pressure, which is done by a *foot-shaped* instrument, it is then and there held by the plugger in the left hand, while with the right hand we are enabled deliberately to make it more perfect by the use of one or more pellets, or by further packing any compressible portion of the cylinder itself. In short, we now substantially *finish* this portion of the filling, so far as the use of the plugger is concerned. It *must* be so left as to have no further occasion to use the plugger upon it. The operation, after this is accomplished in the manner described, is comparatively simple and easy, and may always be reduced to a certainty.

After the upper portion of the filling is completed, a firm compress of wood, paper, rubber, cloth, or any other material, may be carried up between the teeth, even covering in the main this finished portion of the filling to prevent any moisture from above reaching the part yet to be finished.

In this way we may fill the balance of the cavity with the utmost deliberation and ease, and free from all dread of being flooded before the final blow can be struck. This description applies to all lateral and approximal cavities, and indeed in a great measure to all cavities, wherever situated.

My second reason, and one which with me has great weight, is that, take cavities as they average, the work can be done by this method in one-half the time required to make equally good fillings with pellets alone. We sometimes hear dentists boast of spending a great length of time in performing operations, and perhaps on simple fillings, as if there was some peculiar merit in the fact. Should a surgeon boast of spending *hours* upon an operation which could have been as well and as safely done in as many minutes, no one would regard such an admission, not to say boast, as very much to his credit, and I unhesitatingly accuse any dentist who employs more time than is necessary to complete an operation properly, of wrongfully cheating both himself and his patient out of this valuable commodity—*time.*

Again, no dentist has a right to keep his patient in an uncomfortable, not to say painful, attitude one moment longer than necessity positively demands; and as between methods or processes, other things being *equal*, that system should have the preference which secures equally good operations in the shortest space of time. In saying this, I by no means intend to encourage or *countenance* any curtail ment of time that must bring with it any possible shortcoming in the perfection of the operation ; but if one system of operating will materially lessen the time, without marring the result, it can but prove a benefactor to both dentist and patient.

This question of time has not only a strong bearing upon the comfort of both parties, but is of even greater moment when considered as an element of success or failure in performing operations. It not unfrequently occurs that in those cases where the saliva is uncontrollable for any considerable length of time, that the operator is enabled to do the first half of his filling well, and to his entire satisfaction, but fails totally in the last half, and consequently in the whole, by reason of a flood of saliva overtaking him at this point. Now if, by any plan or device, he could have completed his operation "before the flood," he would often avoid such a result, and save himself a panic which unfits him for operating even with ordinary rapidity and success ; and it often occurs where, with ever so dexterous a use of wedge, strings, cofferdams, napkins, etc., this destroying flood submerges both our operations and our hopes, before we can by any possibility get out of its way.

After a practice of nearly a third of a century, I can truly say that any means calculated to avert, or outrun, the evils arising from that rapid flow of saliva which the dentist in many instances has to contend with, should be hailed with a joyous welcome, as relieving him of more than one-half of all the plagues incident to dental operations. But I have already trespassed upon the ordinary limits of such a communication, and far beyond my own intentions when I began this paper—*Dental Cosmos.*

